# The effects of swimming sports on the prevention and restoration of COVID-19 and its variant strains: A protocol for systematic review and meta-analysis

**DOI:** 10.1097/MD.0000000000028571

**Published:** 2022-01-14

**Authors:** Jing Zeng, Qing Liu, Yang Wang, Zhengfang Lei, Yao Shang, Qunru Yang

**Affiliations:** a Chengdu Sport University, Chengdu, Sichuan Province, China; b Chengdu University of Traditional Chinese Medicine, Chengdu, Sichuan, China.

**Keywords:** corona virus disease 2019, delta, omicron, swimming sport, systematic review

## Abstract

**Introduction:**

Since 2019, corona virus disease 2019 (COVID-19) has become a new round of “epidemic,” which has brought about a major crisis to the world from national development, to people's life safety and mental health. Faced with the constant variation of viruses, from COVID-19 to Delta to Omicron. How to curb its further deterioration and enhance human defense against viruses is the focus of scientific researchers. From previous studies, we found that in addition to basic medical treatment, swimming with a certain amount of load and intensity can promote the ventilator of the human body, thereby playing an auxiliary and preventive role in the treatment of COVID-19 and its variant strains.

**Methods:**

This study searched China knowledge network, Web of science, Google scholar, PubMed database to search for the relevant research on swimming prevention and treatment for COVID-19, and the deadline for searching was December 2021. Two researchers independently screened and extracted the literature, and evaluated the bias risk of the included studies. The methodological quality of the included literature was evaluated by the Chochrane bias risk assessment tool.

**Result:**

This study will provide new evidence for the prevention and recovery of COVID-19 and its variant strains by swimming.

**Conclusion:**

To provide a method to help the prevention and restoration of COVID-19 and its variant strains by swimming.

**INPLASY registration number:**

INPLASY2021120075.

## 1 Introduction

Since the major crisis of corona virus disease 2019 (COVID-19) on global economy, life and health in 2019, with the constant variation of viruses, Delta and Omicron have been produced in succession, From past cough, fever and other related symptoms to asymptomatic infection, their transmission speed and infectiousness are far more than the harm brought by COVID-19. The novel corona virus pneumonia announced in March 2020 that WHO threatened the world's public health, national economy, security and social relations and international relations. By systematically combing the academic literature of journals at home and abroad since the outbreak of the epidemic, scholars’ research is roughly divided into 3 aspects: first, epidemiological research, mainly through building models, carrying out systematic analysis, summarizing the transmission routes of the virus and predicting the trend of the epidemic, so as to effectively control the spread of the disease.^[1]^ Tang^[2]^ estimated the basic reproduction number of virus by constructing a mathematical model, which can be used to measure the possibility and severity of epidemic outbreak, and provide key information for determining the type and intensity of disease intervention. Chan^[3]^ carried out a traceable study of COVID-19, trying to explain the reasons why beta COVID-19 mutation is fast and the host is more. Huang^[4]^ summarized COVID-19 patients’ epidemiology, clinical, laboratory and radioactive features, treatment and clinical results. Lv^[5]^ was found that Chinese medicine played a certain role in the prevention and treatment of COVID-19. Xue^[6]^ analyzed the role of Chinese medicine that is good for lung in epidemic prevention and control and clinical treatment from the perspective of traditional Chinese medicine theory. Different scholars have studied COVID-19 from different perspectives, and put forward corresponding treatment plan. However, so far, the relevant research on COVID-19 mainly focuses on traditional medical research, and the current medical research also includes the assistance and recovery of COVID-19 by traditional Chinese medicine. As for sports promoting health, it is still relatively weak to analyze how to prevent COVID-19 from the perspective of sports and the recovery after curing COVID-19. Among many sports, the improvement of human lung function by swimming has been recognized all over the world. Therefore, through swimming to improve lung function, so as to achieve the prevention of infection with COVID-19 and the recovery effect after the cure, is conducive to the COVID-19 and its variants by people all over the world. Therefore, COVID-19 and its variant strains and their variants were systematically analyzed by systematic evaluation in order to provide new evidence for the treatment and recovery of new crown pneumonia and its variant strains.

## 2 Methods

### 2.1 Registration

This study protocol systematic review has been registered in INPLASY. The registration number is INPLASY2021120075.

### 2.2 Inclusion criteria

#### 2.2.1 Study designs

We will include researches related to Swimming Sports of patients suffering from COVID-19.Studies will be selected according to the criteria outlined below:

1. **Study type**: Randomized controlled trial.

#### 2.2.2 Participants

The people who were confirmed to have COVID-19 (positive nucleic acid test);

#### 2.2.3 Intervention measures

The experimental group received medical treatments and Swimming Sports intervention scheme after medical treatments; The control group only received the medical treatments

#### 2.2.4 Outcome measures

**Primary outcomes:** Nucleic acid test

**Secondary outcomes:** fever, cough, Nucleic acid test, temperature recovery time.

#### 2.2.5 Exclusion criteria

Review and comment research or nonChinese and English literature; In the study, only the experimental group, no control group or the control group is the literature of blank control; Literature published in the form of abstracts, research that cannot obtain the full text, or literature with incomplete research data and unsuccessful contact with the author.

### 2.3 Data sources

According to the PICOS principle, the second and third authors of this paper searched CNKI, web of science, WanFang Data, Google Scholar and Sport discussion databases by computer to collect relevant research on the effects of Swimming Sports on the treatment of COVID-19. The retrieval time limit is from the establishment of the database to February 2021.

### 2.4 Data collection and analysis

#### 2.4.1 Search strategy

In addition, the references of the retrieved literature are traced to supplement the relevant literature. The retrieval adopts the combination of subject words and free words. The Key words are “Swimming Sports,” “COVID-19,” “Corona Virus Disease 2019,” “Novel Corona Virus,” “019-nCoV” et al; Taking CNKI search library as an example, the specific search strategy of this study is shown in Box 1. The same strategies are used in other electronic databases.

**Box 1 CNKI searching**

#1 Swimming
#2 Swimming Sport
#3 COVID-19
#4 Corona Virus Disease 2019
#5 Delta
#6 Omicron
#7 Swimming or COVID-19 or Corona Virus Disease 2019
#8 Swimming or Delta
#9 Swimming or Omicron
#10 Swimming and COVID-19 or Corona Virus Disease 2019
#11 Swimming and Delta
#12 Swimming and Omicron

#### 2.4.2 Study selection

**Figure 1:**
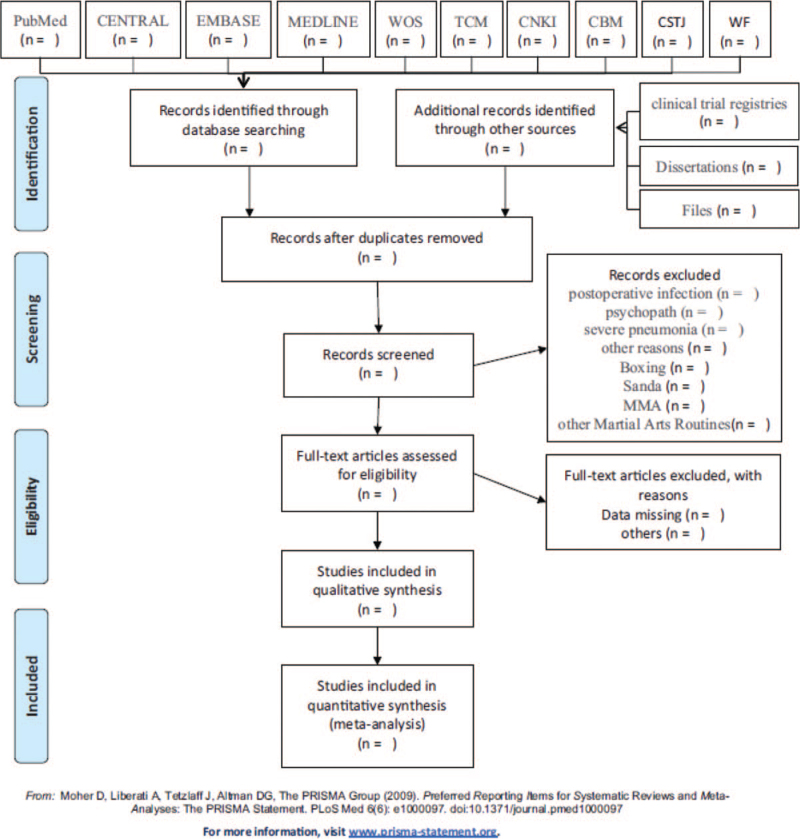
Flow chart of the study.

The literature were retrieved from domestic and foreign databases, including China Knowledge Network (CNKI), web of Science and Google Scholar. At the same time, it is supplemented by literature review. Finally, Endnote X9 was used to search the retrieved documents and eliminated duplicate documents; If it fails to contact the author of the literature whose full text cannot be obtained, it will be eliminated; Then, the literature is eliminated by reading the title and abstract; Finally, the remaining literature was rescreened by full-text reading, and the review literature, literature with no data or incomplete data, literature with inconsistent outcome indicators and literature without control group were eliminated. The process of literature screening is shown in Figure 1. The basic data of the finally included literature are extracted. The extraction indicators include: author, year of publication, sample size, intervention scheme, frequency and outcome indicators.

#### 2.4.3 Measures of effect

The effect quantity of meta-analysis can be combined into “random effect model” and “fixed effect model.”^[7]^ The difference is that the random effect model assumes that there are differences between samples, and its results are more generalized; The fixed effect model assumes that the mean values of samples are the same, and its results are not suitable to be extended to studies not included in meta-analysis. At the same time, this study uses the Review Manager Version 5.4 software to analyze the data of the included literature. The measurement data uses the mean standard deviation to analyze the effect quantity, and the confidence interval is 95% confidence interval.

#### 2.4.4 Data extraction

Two researchers (the second author and the third author) separately searched, screened, confirmed and included the literature according to the inclusion and exclusion criteria. In case of different opinions, discuss and solve them with the first author of this paper. In the first screening of literature, the main way is to exclude the research that is obviously irrelevant to the research topic through reading the topic; Then, by further reading the abstract and the full text, it is clear whether the article is Randomized controlled trial; Whether the data is complete, etc. In case of failure to obtain complete data or information, contact the author for further confirmation. At the same time,

1. the data of “first author,” “publication time,” “intervention mode,” “utility” and so on are extracted;
2. Extract the key elements of bias risk assessment;
3. Extract outcome indicators and data.

#### 2.4.5 Risk of bias assessment

Publication bias means that in the process of publication, statistically significant positive research results are more likely to be published than those non-statistically significant negative research results. It includes author bias, editor bias, language bias, research fund source bias and so on. Since meta-analysis is a quantitative evaluation of the literature, the literature with negative research results should be included in the analysis of the literature.^[8]^ However, because it is difficult to publish, there are some difficulties in collection, which may affect the results of META analysis to a certain extent. Therefore, when conducting meta-analysis, researchers need to use relevant methods to test the publication bias of the finally included literature. The funnel chart method is used in this study, which is also a commonly used method to test publication bias.22.

#### 2.4.6 Dealing with missing data

Dealing with missing data. In order to ensure the integrity and accuracy of the data, when the data is missing, we will contact the corresponding author to try to obtain the complete data. If the corresponding author cannot be contacted or the corresponding author does not reply, we can only delete incomplete data to ensure the accuracy of the study.

#### 2.4.7 Data synthesis

Heterogeneity analysis test is to test various potential intra group and inter group differences and different qualitative conditions that affect the effect quantity. The usual test method is *I*^2^ or *q* test. In this study, *I*^2^ statistic is used for test, and the test formula is: *I*^2^ = (Q–[k–1]/q)×100%. When *I*^2^ < 50%, *P* ≥ .1, it is considered that there is no obvious heterogeneity among various studies. At this time, the fixed effects model is used to calculate and combine the effects; If *I*^2^ > 50%, *P* < .1, it is considered that the heterogeneity is high. At this time, further heterogeneity source analysis will be carried out. Generally, subgroup analysis or sensitivity analysis will be used, and the literature will be eliminated one by one to observe the heterogeneity, and random effects model will be used to merge the effects.^[7]^ However, the Cochrane reviewers’ Handbook believes that comparing the differences between subgroups is indeed helpful to understand the regulatory variables, but the essence of subgroup analysis is observational, and it does not strictly control other interfering variables. Therefore, subgroup analysis has certain limitations. When conducting subgroup analysis, the conclusion of subgroup effect should be carefully explained, finally, *P* value combined with 95% confidence interval was selected as the effect quantity for statistical analysis. In addition, according to Cohen,^[9]^ 0.2 is a small effect quantity; 0.5 is the medium effect quantity; 0.8 is a large effect.

#### 2.4.8 Subgroup analysis

According to the results of meta-analysis and the purpose of this study, subgroup analysis of regulatory variables was carried out to further explore the source of heterogeneity and compare the effect amount between subgroups.

#### 2.4.9 Sensitivity analysis

Sensitivity analysis is used to analyze research quality, intervention method, publishing type, and so on. If there is large heterogeneity, sensitivity analysis should be carried out. The sensitivity analysis adopts the method of eliminating the literature one by one.

#### 2.4.10 Grading the quality of evidence

The risk of bias included in the study was independently evaluated by 2 researchers through the Cochrane manual 5.1.0 bias risk assessment tool.^[10]^

## 3 Discussion

This article mainly introduces how to systematically analyze the prevention and recovery of COVID-19 from swimming in the aspects of literature download process, screening and inclusion criteria, data collection and analysis, heterogeneity analysis, sensitivity analysis and subgroup analysis. However, the article has some limitations, such as no analysis of nonEnglish literature, and the sample size of literature screening needs to be further expanded.

## 4 Limitations

Due to the extremely variant strain of COVID-19, a virus for the whole world, we should pay attention to nonEnglish language analysis in order to ensure the accuracy of the conclusion, so that other scholars can further study COVID-19.

## Acknowledgment

Jing Zeng is the first author.

## Author contributions

**Conceptualization:** Jing Zeng.

**Data curation:** Yang Wang, Qunru Yang.

**Formal analysis:** Jing Zeng, Yao Shang.

**Funding acquisition:** Zhengfang Lei.

**Methodology:** Qing Liu, Zhengfang Lei.

**Software:** Yang Wang, Yao Shang.

**Writing – original draft:** Jing Zeng.

**Writing – review & editing:** Qing Liu.
